# The Sodium Priority Hypothesis: A Conceptual Taste–Nutrition Framework Linking Salt Taste, Umami and Food Acceptance

**DOI:** 10.3390/nu18172914

**Published:** 2026-09-04

**Authors:** Russell Keast, Andrew Costanzo, Claudia Hartley, Lynn Riddell

**Affiliations:** 1 Deakin Centre for Advanced Food Sciences, School of Exercise and Nutrition Sciences, Deakin University, Burwood, VIC 3125, Australia; andrew.costanzo@deakin.edu.au (A.C.);; 2 ARC Training Centre for Hyphenated Analytical Separation Technologies (HyTECH), Deakin University, Burwood, VIC 3125, Australia; 3 Faculty of Health, Deakin University, Burwood Highway, Burwood, VIC 3125, Australia

**Keywords:** sodium, salt taste, chloride, umami, monosodium glutamate, bitterness suppression, sodium appetite, food acceptance, sodium reduction 35

## Abstract

Taste contributes to food selection by integrating sensory information with physiological state and prior experience. Sodium is essential, continually lost, and homeostatically regulated, yet it is abundant and frequently overconsumed in contemporary food environments. This conceptual narrative review proposes the Sodium Priority Hypothesis, as follows: sodium may function as a nutritional target whose acquisition and food acceptance are supported by several partly independent sensory routes. Sodium chloride provides the dominant dietary route to saltiness, although the molecular basis of human salt taste remains unresolved and may involve both sodium- and chloride-dependent mechanisms. Sodium salts can also suppress bitterness and modify taste mixtures in concentration- and matrix-dependent ways. A further, more speculative proposition is that sodium-bearing umami stimuli may have behavioural relevance beyond glutamate-derived umami quality. The framework is intended to connect two timescales: the physiological and evolutionary pressures that promoted sodium acquisition when availability was variable, and the contemporary food environment, in which abundant, inexpensive sodium is widely used to enhance food palatability. It does not claim that sodium alone explains modern excess intake, that prehistoric populations routinely added salt, or that sodium is required for activation of umami receptors. Evidence is strongest for sodium physiology, sodium appetite, salt taste, and selected mixture effects; direct evidence for a sodium-specific contribution to umami liking or intake remains limited. The hypothesis is therefore an organising and falsifiable framework that requires experimental validation. It is explanatory rather than prescriptive.

## 1. Introduction

Taste is a biological system that helps humans and other animals evaluate foods in relation to nutrient availability, potential harm, physiological state, and prior experience [1]. Conventional associations, such as sweetness with carbohydrate, bitterness with potentially harmful compounds, sourness with acidity, saltiness with mineral balance, and umami with foods containing glutamate, are useful but incomplete. Taste qualities are not direct nutrient labels; they are components of a broader sensory system that integrates taste with aroma, texture, temperature, chemesthesis, and post-ingestive feedback.

Sodium is the major extracellular cation and is required for extracellular fluid volume, osmotic regulation, nerve transmission, muscle contraction, and nutrient transport [2]. It is continually lost in urine, sweat, and faeces and must be replaced through the diet. Sodium depletion can generate a specific motivational state known as sodium appetite, a response supported most strongly by animal studies and less consistently by human studies [3,4,5]. In contemporary diets, sodium chloride is inexpensive, widely incorporated into manufactured and restaurant foods and commonly consumed above recommended levels. High sodium intake contributes to elevated blood pressure and cardiovascular risk, creating a marked contrast between physiological need and modern exposure [6,7,8].

Existing studies in the literature explain important parts of this system separately. Homeostatic models explain sodium need and appetite; salt-taste research examines peripheral and central detection; umami research addresses glutamate and nucleotide signalling; and food-sensory research examines flavour integration and acceptance (Table 1). What is less developed is an organising framework asking whether several sodium-associated sensory effects converge on the acceptance or acquisition of sodium-containing foods. The Sodium Priority Hypothesis addresses this gap. It proposes, rather than demonstrates, that sodium may operate as a nutritional target whose acquisition is supported by multiple partly independent routes: NaCl saltiness, selected taste mixture effects, and sodium-associated savoury signals [3,4,5,9,10,11,12,13,14]. “Priority” refers to this proposed integrative nutritional relevance; it does not mean that sodium dominates all nutrients, all taste mechanisms, or all food choices.

This framework addresses two related but distinct questions. First, under conditions in which sodium availability was variable, could sensory and motivational systems that promoted sodium acquisition have been advantageous? Second, in modern food environments, could those systems make sodium reduction difficult when sodium is embedded in palatable foods? The hypothesis is not intended to reconstruct a universal prehistoric diet or to explain an insatiable modern appetite for salt. Modern overconsumption also reflects availability, food processing, learned preferences, cuisine, portion size, and product structure. This framework is therefore a mismatch hypothesis with potential relevance to sodium reduction, not a recommendation for greater sodium intake [6,7,8,9,10].

**Table 1:** Proposed sensory routes within the Sodium Priority Hypothesis, with evidence status and boundary conditions.

Proposed Route	Established Sensory Observation	Proposed Relevance to Sodium Acquisition or Acceptance	Evidence Status	Key References
NaCl salt-taste route	NaCl produces the dominant familiar salty percept; concentration affects liking and aversion.	Provides a direct sensory route to sodium containing foods. Human molecular transduction remains unresolved.	Established percept; species-dependent mechanism.	[9,11,12,15,16,17,18]
Counter-ion modulation	Sodium salts differ in saltiness, sourness, bitterness, alkalinity, and temporal profile according to the accompanying anion and matrix.	Different counter-ions alter how sodium is sensorially delivered.	Established; food matrix comparisons remain limited.	[9,18,19]
Taste-mixture modulation	NaCl can suppress selected bitter responses and alter sweet, sour, and umami mixtures.	May alter the acceptability of some sodium-containing foods.	Established but stimulus-, concentration-, and matrix-dependent.	[9,20,21,22,23,24,25]
Sodium-linked umami	MSG provides glutamate-derived umami while also containing sodium.	Defines a testable behavioural comparison.	Prediction only; direct comparative evidence is limited.	[26,27,28,29,30,31,32,33,34]
Ribonucleotide-associated amplification	Inosinate and guanylate amplify glutamate derived umami; commonly used flavour enhancers are often sodium salts.	Defines a further testable comparison of sodium-containing versus non-sodium nucleotide systems.	Umami synergy established; sodium-specific behavioural contribution remains a prediction.	[26,27,33,34]
Food-matrix and learning pathways	NaCl affects texture, water binding, volatile release, preservation, and learned flavour expectations.	Can alter acceptance independently of a sodium-specific taste mechanism.	Established boundary conditions; not evidence of sodium priority by themselves.	[9,10,35]

### Purpose and Scope of the Review

This conceptual narrative review integrates evidence from sodium physiology, sodium appetite, salt-taste reception, counter-ion effects, taste-mixture interactions, umami perception, food acceptance, and sodium reduction. The primary focus is human taste perception and food acceptance. Rodent and other animal studies are included when they provide mechanistic information about sodium appetite, taste transduction, or neural regulation that cannot be obtained readily from human studies. This review distinguishes demonstrated observations, integrative inference, and untested prediction. It does not attempt a comprehensive review of renal physiology, cardiovascular epidemiology, food microbiology, or all the technological functions of salt; these are considered where they define relevant physiological or food-matrix boundaries. Terminology is used according to context throughout: “sodium” refers to the nutrient, dietary exposure or physiological state; “Na+” or “sodium ion” is used for ionic or receptor-level mechanisms; and “NaCl” or the specific sodium salt is used when a defined chemical stimulus is meant.

## 2. Literature Review Approach

Targeted searches were conducted in PubMed, supplemented by targeted searches of journal publisher websites, reference list searching, and forward citation searching, for peer-reviewed English-language studies available through July 2026. Search combinations included “sodium appetite”, “salt taste”, “NaCl taste receptor”, “ENaC taste”, “amiloride salt taste”, “chloride taste”, “TMC4”, “sodium salt anion effect”, “sodium bitterness suppression”, “salt sweetness interaction”, “salt sourness interaction”, “monosodium glutamate”, “umami discrimination”, “glutamate salt”, “ribonucleotide synergy”, “sodium reduction”, “food matrix saltiness” and related terms. Searches were iteratively refined because the objective was conceptual integration rather than estimation of a single intervention effect.

Eligible evidence included human sensory and psychophysical studies, human depletion or exposure studies, observational studies relevant to intake or discrimination, animal studies of sodium appetite and neural regulation, receptor or cellular studies, and food-matrix studies directly relevant to the proposed framework. Studies were included when they directly informed one or more of the review questions, provided empirical or mechanistic evidence relevant to the framework, or helped define an alternative explanation or boundary condition. Seminal studies were retained when they provided original evidence not replaced by newer work. Non-peer-reviewed commentary, duplicate reports, and studies without a clear connection to sodium sensing, sodium-related food perception or acceptance, or the defined boundary conditions were not considered in detail. Study selection and synthesis were author-led and interpretive, as appropriate for a conceptual narrative review, but were criterion-guided rather than arbitrary or algorithmic. No automated screening software, formal risk-of-bias tool, or meta-analysis was used. The aim was transparency in how evidence was selected and interpreted, rather than the exhaustive study identification and reproducibility expected of a systematic review.

Evidence was evaluated against five questions: (1) whether sodium has a distinctive physiological or motivational role; (2) what is known about human versus animal salt-taste mechanisms; (3) whether sodium changes food perception beyond saltiness; (4) whether sodium-bearing umami stimuli have behavioural effects that cannot be explained by glutamate quality, food structure, or learning alone; and (5) whether the integrated proposition yielded predictions that could be experimentally supported or rejected.

## 3. Sodium Physiology, Sodium Appetite, and Historical Context

Sodium homeostasis is regulated by renal, endocrine, and neural mechanisms. Renin–angiotensin–aldosterone signalling, vasopressin, natriuretic pathways, and central circuits influence sodium conservation, thirst, and sodium appetite [2,3,4,5,13,14]. These systems define the physiological need state; the present hypothesis concerns the sensory and behavioural routes through which sodium-containing sources may then be detected and accepted. Recent animal studies demonstrate that internal sodium state can alter the valence of salt and have identified gut–brain and medulla–hypothalamic pathways that contribute to sodium appetite [13,14]. Equivalent causal evidence in humans is more limited.

The evolutionary argument requires caution. Terrestrial sodium availability has varied markedly by ecology and subsistence pattern, and it should not be inferred that all ancestral or traditional populations routinely added salt to food. The Yanomami population studied by Oliver and colleagues consumed no discretionary salt, exhibited extremely low urinary sodium excretion, and maintained sodium balance through strong sodium-conserving hormonal responses [36]. Reviews also document traditional populations with substantially lower sodium intakes than industrialised populations [6]. Conversely, other societies collected, produced, or traded mineral or plant-derived salts. These observations show variability rather than a single prehistoric pattern. They are compatible with the narrower proposition that mechanisms conserving sodium and motivating its acquisition could have been useful when sodium was limited. They do not demonstrate universal salt seeking or culinary salt use.

Controlled sodium depletion in humans has produced modest changes in salt preference and the attractiveness of salty foods, whereas animal depletion studies show more robust changes in NaCl ingestion and gustatory responses [37,38]. Animal findings are used here as mechanistic context and are not treated as evidence that an equivalent human response has been demonstrated. Thus, evidence that physiological sodium state can influence sodium seeking is strong in animals and suggestive but heterogeneous in humans. Most studies have used NaCl rather than MSG, nucleotide salts, or complex foods. Whether sodium appetite generalises across these chemically and sensorially distinct sources remains unknown and is a central test of the hypothesis.

Salt valence is concentration- and state-dependent. Moderate NaCl concentrations can be acceptable or preferred, whereas high concentrations become aversive and depletion can shift this valence in animal models [11,12,13]. This pattern is consistent with regulated ingestion but does not imply that taste alone prevents excess intake in a modern environment where sodium is widely distributed across foods and repeatedly learned in palatable contexts.

## 4. Salt Taste Reception: Sodium, Chloride, and Species Differences

Any sodium specific hypothesis must distinguish the nutritional identity of sodium from the molecular transduction of the NaCl percept. In rodents, low-concentration appetitive salt responses depend substantially on an amiloride sensitive epithelial sodium channel (ENaC) pathway, while higher salt concentrations recruit additional amiloride-insensitive and -aversive pathways [11,12]. These results provide strong evidence for sodium selective peripheral detection in mice, but they should not be transferred uncritically to humans.

The mechanism of human salt taste transduction remains unresolved. ENaC subunits, including the human δ subunit, have been identified in human taste tissue, yet psychophysical effects of amiloride are smaller and less consistent than in rodents [15,17]. Human salt perception is therefore thought to rely substantially on amiloride-insensitive mechanisms. Recent work has renewed interest in chloride; TMC4 functions as a chloride channel and Tmc4-deficient mice show reduced responses to high concentration chloride salts [15,16]. Human psychophysical studies also show that sodium salts with different anions differ in saltiness and temporal response [18,19]. However, current evidence does not establish that human NaCl detection is mediated by chloride rather than sodium, or by a single chloride specific pathway. The most defensible conclusion is that NaCl perception may reflect multiple ion- and concentration-dependent mechanisms whose relative contribution differs by species.

This uncertainty does not require changing the name of the Sodium Priority Hypothesis. The title refers to sodium as the proposed nutritional target and to the behavioural consequences of sodium-containing foods, not to a claim that Na+ alone activates every receptor responsible for saltiness. The framework explicitly recognises that chloride and other counter-ions shape the sensory expression of sodium and that molecular sodium specificity in humans remains an important boundary condition.

## 5. Sodium Versus Potassium: Why Not a General Cation Appetite?

Potassium is essential and is the major intracellular cation, so essentiality alone cannot establish sodium priority. Potassium is generally abundant in unprocessed plant foods, although availability varies across diets. KCl can produce saltiness but usually has weaker salty quality and bitter or metallic side notes, especially as substitution increases [9,20]. In model chicken broths, sodium but not potassium suppressed bitterness [20]. These observations make potassium a useful comparator, but they do not prove a sodium-specific evolutionary pathway.

The molecular basis of potassium taste is also not fully resolved and may involve broadly tuned amiloride-insensitive salt pathways together with bitterness and other side-taste mechanisms. Human sensory differences between NaCl and KCl demonstrate distinct perceptual profiles, not necessarily a single receptor that identifies nutritional sodium. The hypothesis therefore predicts a behavioural distinction that must be tested under matched saltiness, bitterness, concentration, and food-matrix conditions.

## 6. Counter-Ions as Sensory Modifiers

Dietary sodium is present predominantly as Na+ paired with counter-ions rather than as elemental sodium or an isolated cation. Chloride, glutamate, citrate, phosphate, bicarbonate, acetate, lactate, and other counter-ions influence saltiness, sourness, alkalinity, bitterness, metallic notes, temporal profile, and overall acceptability [9,18,19]. NaCl produces the most familiar salty percept, whereas sodium citrate may contribute sour, salty, and buffering effects; sodium acetate can introduce sour or vinegar-like qualities; sodium phosphates may produce saline, alkaline, or metallic notes; and sodium bicarbonate is alkaline and can be bitter at higher concentrations [9,18,19]. These properties vary with concentration, pH, and matrix.

The term “sensory vehicle” is therefore used cautiously. Counter-ions do not merely carry sodium, they participate in perception and may alter the route by which a sodium-containing compound is recognised or accepted. Recent human work with citric acid and citrate salts further demonstrates that the number and identity of counter-ions can influence sourness, saltiness, and oral sensations [19]. This chemistry is consistent with an integrative framework but is not evidence that the sensory system extracts a pure sodium signal from every salt.

## 7. Sodium Chloride as the Dominant Salt-Taste Route

NaCl is soluble, stable, widely available and highly effective in producing saltiness. It is central to seasoning, preservation, and many structural functions in food [9,10]. Under the hypothesis, NaCl is described as the dominant dietary and culinary route to saltiness, not as proof that chloride is merely passive or that Na+ is the sole transduced ion. Its perceptual clarity probably reflects the combined behaviour of sodium, chloride, and tissue pathways, with species- and concentration-dependent contributions [15,16,17,18].

Other sodium salts may be less salty or introduce sour, bitter, metallic, alkaline, or savoury qualities. This helps explain the culinary dominance of NaCl, but the hypothesis does not require all sodium compounds to be liked. Instead, it asks whether the sensory properties of selected sodium salts, particularly NaCl, create effective routes to detecting and consuming sodium within specific ecological and food contexts.

## 8. Sodium as a Flavour Modulator in Foods

NaCl has perceptual effects beyond saltiness. It can suppress the bitterness of selected compounds, alter sweet and sour perception, interact with umami, and change overall flavour balance [9,20,21,22,23,24,25]. These effects are not universal. Their direction and magnitude depend on the bitter stimulus, relative taste intensities, concentration, pH, counter-ion, adaptation, individual differences, and the food matrix. Human mixture studies often show suppression of taste qualities rather than a simple enhancement of all desirable tastes [25].

Bitterness suppression is one of the clearest examples. Sodium salts reduce the bitterness of quinine, caffeine, magnesium salts, and KCl in some conditions [20,21,22,23]. Receptor assays indicate that NaCl reduces activation for selected human bitter receptor–agonist pairs but not others, suggesting both peripheral and central contributions [24]. Thus, sodium-mediated bitterness suppression is a genuine but stimulus-specific mechanism and it should not be generalised to all bitter foods or all concentrations.

Salt–sweet and salt–sour interactions broaden the sensory context of the framework but are not proposed as independent sodium-acquisition mechanisms. Rather, they demonstrate that NaCl can alter the perceptual balance of taste mixtures beyond saltiness itself. Depending on stimulus combination, relative intensity, pH, and matrix, NaCl may enhance, suppress, or have little effect on sweetness or sourness, while acids and citrate salts can alter salt detection and oral sensations [19,25]. Within the Sodium Priority Hypothesis, these interactions are relevant only insofar as their net effect changes the acceptance or choice of a sodium-containing food. They do not imply that every sodium-related interaction promotes intake.

The evolutionary interpretation does not propose that prehistoric people routinely added salt to bitter plants. Direct evidence for such discretionary use across prehistoric populations is unavailable and ethnographic practices are heterogeneous. A possibility is that when sodium co-occurred with a food or environmental source, mixture effects could have increased acceptance of the food. The strongest evidence and clearest relevance for deliberate bitterness suppression are in modern culinary practices and manufactured foods.

### Food-Matrix and Processing Boundary Conditions

Reducing or replacing NaCl changes foods through more than receptor-level taste. Salt can influence protein solubilisation, water binding, gelation, viscosity, texture, fermentation, microbial stability, and the generation or release of volatile compounds [10,35]. A reduced-sodium food may therefore be less acceptable because of altered texture, aroma, or shelf stability rather than because a sodium-specific oral signal has been removed. Conversely, structural design can increase sodium release and perceived saltiness without increasing total sodium.

These indirect effects are important alternative explanations. Experiments intended to test the Sodium Priority Hypothesis should use simple solutions when isolating receptor or mixture mechanisms and carefully matched food matrices when testing acceptance. Instrumental texture analysis, volatility analysis, microbiological stability assessment, and temporal sensory methods may be required to separate sodium taste from product-structure effects.

## 9. Umami, Monosodium Glutamate, and the Proposed Sodium-Linked Savoury Route

Umami is generated primarily through glutamate-sensitive receptor pathways. The heterodimeric class C G-protein-coupled receptor T1R1/T1R3 is widely recognised as a principal mammalian umami receptor, while taste-variant metabotropic glutamate receptors have also been proposed to contribute; 5′-ribonucleotides enhance glutamate-derived umami through receptor-level synergy [26,27]. These pathways are mechanistically distinct from salt-taste transduction and from bitter-taste signalling through TAS2R receptors [24]. The sodium ion is therefore not required in order to explain activation of the umami receptor system. As noted by Kurihara, MSG–nucleotide neural synergy can be amiloride-insensitive, demonstrating that the large synergistic response is not simply an Na+ response [26]. The sodium-priority perspective raises a separate behavioural question: whether a sodium-containing umami stimulus influences liking, reinforcement, or intake differently from a perceptually matched non-sodium umami stimulus. Convergence at a behavioural or hedonic level does not imply a shared peripheral receptor mechanism.

The ecological interpretation of umami also requires restraint. In many fresh protein-rich foods, most glutamate is protein-bound rather than freely available to activate oral umami receptors. Across unprocessed foods, free glutamate concentration is not a reliable proxy for total protein content [28,29]. Free glutamate becomes more prominent in selected ripe vegetables, seaweeds, and foods that are aged, fermented, hydrolysed, or cooked. Consequently, the sodium-linked umami route has clear relevance to modern culinary and manufactured foods, but its adaptive importance in prehistoric human diets is uncertain and should be treated as speculative rather than foundational.

MSG remains useful as an experimental compound because it combines glutamate-derived umami with sodium in a defined stimulus. Human sodium-matched discrimination methods show that individuals differ in their ability to distinguish MSG from NaCl, and discrimination status has been associated with salt-recognition thresholds and selected dietary behaviours [31,32]. These cross-sectional associations do not establish causality or sodium priority, but they demonstrate that glutamate quality and sodium-related saltiness can be studied as separable components. Whether sodium-bearing umami stimuli have different hedonic or ingestive consequences from appropriately matched non-sodium umami stimuli remains an unresolved prediction and is considered under Testable Predictions and Future Research Priorities (Section 15).

### Ribonucleotides and Umami Amplification

Inosinate and guanylate amplify glutamate-derived umami, and commonly used flavour enhancers include disodium inosinate and disodium guanylate [26,27,33,34]. The umami synergy itself is well established and does not depend on sodium transduction. Within the Sodium Priority Hypothesis, sodium-containing forms are not treated as evidence of sodium priority; they identify a further testable comparison between sodium-containing and non-sodium nucleotide systems, considered in Section 15.

## 10. Critical Synthesis: Established Evidence, Inference, and Gaps

The framework is strongest where independent evidence converges on sodium as a distinctive physiological and sensory variable: essentiality, regulated loss, depletion-induced sodium appetite, concentration- and state-dependent salt valence, the distinctive sensory profile of NaCl, selected bitterness suppression, and the practical difficulty of replacing salt in foods. It is weaker where the argument moves from co-occurrence to sodium-specific causation. In particular, sodium-linked umami and an integrated evolutionary outcome remain hypotheses. Table 2 separates evidence type, consistency, relevance, and limitation.

## 11. Relationship to Existing Frameworks and Novelty

The Sodium Priority Hypothesis is not intended to replace established models. Its novelty is organisational as it links homeostatic need, salt-taste perception, mixture effects, counter-ion chemistry, food acceptance, and modern sodium reduction challenges in one set of testable propositions (Table 3).

## 12. The Sodium Priority Hypothesis: Integrated Framework

**Figure 1:**
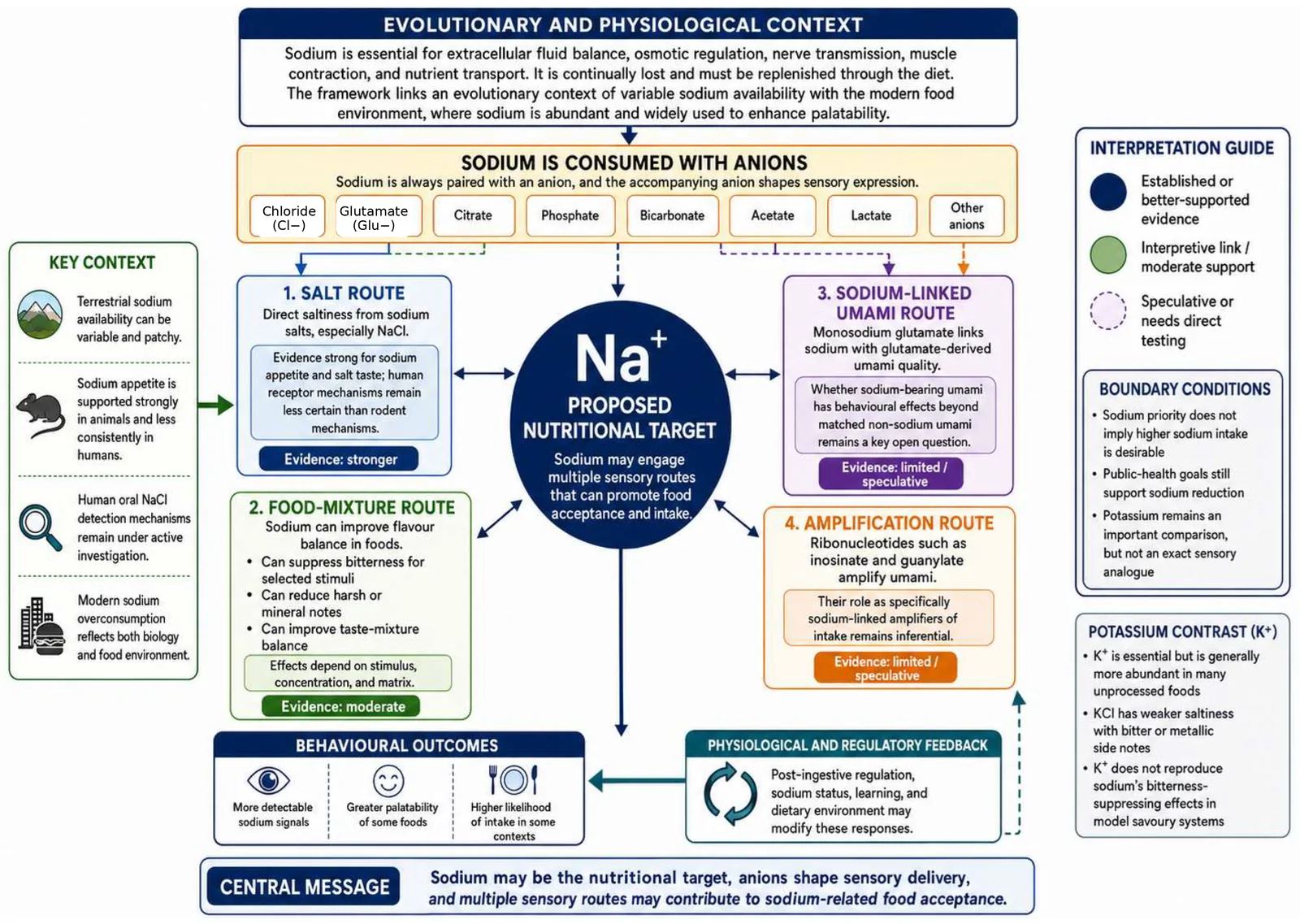
Conceptual framework for the Sodium Priority Hypothesis. Original conceptual figure created by the authors for this manuscript; no previously published figure has beenb reproduced or adapted. The figure distinguishes established physiological and sensory observations from proposed integrative links. Na+ denotes the sodium ion; elsewhere, “sodium” refers to the nutrient or dietary exposure unless a specific sodium salt is named. The evolutionary context does not assume universal discretionary salt use, and the modern food environment pathway does not imply that sodium priority alone explains excess intake.

The framework has four layers (Figure 1). First, sodium is an essential and regulated nutrient, and internal sodium state can modify salt motivation and valence. Second, NaCl provides the dominant dietary route to saltiness, although human transduction may involve sodium- and chloride-dependent mechanisms. Third, sodium salts participate in selected mixture effects that may change food acceptance. Fourth, the framework identifies a putative sodium-linked umami route. This is explicitly a testable prediction rather than supporting evidence and is addressed in Section 15.

Two contexts constrain the model. The evolutionary context is variable sodium availability, not a claim of universal salt use. The modern context is abundant, inexpensive sodium embedded in learned and technologically complex foods. The hypothesis is not restricted to modern societies, rather, it proposes a biologically grounded sodium acquisition framework whose behavioural expression can be modified by culture, learning, and modern food processing. The framework predicts that these established and proposed routes may converge on sodium-containing food acceptance, but it must outperform alternative explanations based on saltiness alone, food structure, exposure, cuisine, and availability. Solid pathways in Figure 1 denote established or directly demonstrated relationships, dashed pathways denote proposed, indirect, or insufficiently tested links.

## 13. Taste–Nutrition Interface: From Detection to Physiological Regulation

Taste signals contribute to food selection and the initiation of ingestion, while internal state and post-ingestive feedback alter motivation and valuation. Animal studies have identified neural and gut–brain pathways that regulate sodium appetite and rapidly modify salt valence [13,14]. The hypothesis places oral NaCl and sodium bearing food cues within this wider system, but it does not claim that oral taste alone regulates systemic sodium balance.

Taste-related receptors and nutrient-sensing pathways are expressed beyond the oral cavity, and gastrointestinal feedback can influence appetite and learning [30]. Direct evidence associating sodium-linked umami cues to human electrolyte regulation, fluid intake, or gut feedback is currently sparse. An oral sodium cue, a glutamate cue, and a post-oral sodium consequence should therefore be treated as separable components until experiments demonstrate functional integration. Oral exposure to NaCl without ingestion, for example, did not alter salt-taste function in a controlled human crossover study [39].

## 14. Alternative Explanations, Conflicting Evidence, and Boundary Conditions

Several findings could support explanations other than sodium priority. Human sodium appetite is less robust and less consistently demonstrated than animal depletion responses. Familiarity, cuisine, and repeated exposure strongly shape preferred salt levels. Gradual sodium reduction can preserve acceptance, showing that liking is adaptable rather than fixed. Modern excess intake may result mainly from product availability, habitual consumption, and sodium distributed across foods, without requiring an unusually strong conscious appetite for salt. NaCl-specific liking can also reflect perceptual quality rather than nutritional targeting. KCl and non-sodium glutamate salts have side tastes that make them imperfect controls. MSG acceptance depends on concentration and congruence with a savoury food matrix. Improved liking of an MSG-containing food cannot isolate a sodium effect, because glutamate, saltiness, aroma, and learned savoury expectations change together.

The chloride debate further limits a simple sodium detection account. Evidence for TMC4 and anion effects shows that NaCl perception cannot be reduced to Na+ passage through a single channel, particularly in humans [15,16,17,18,19]. Similarly, ethnographic evidence shows that humans can maintain sodium balance without discretionary salt [36], and food-composition evidence shows that free glutamate is not a reliable protein signal in natural foods [29]. These observations do not invalidate the hypothesis, but they narrow it to an integrative behavioural proposition rather than a universal receptor or prehistoric-diet theory.

Falsification is possible. The hypothesis would be weakened if sodium-depleted participants showed no greater response to sodium-containing than matched non-sodium stimuli, if MSG, potassium glutamate, and other glutamate salts produced equivalent liking and reinforcement after side tastes were controlled, or if food acceptance were explained fully by saltiness, texture, aroma, learning, and availability without an additional sodium-related contribution.

## 15. Testable Predictions and Future Research Priorities

The priority is not to accumulate further correlations but to conduct discriminating experiments. Randomised sodium-depletion and -repletion studies in humans should compare NaCl with sodium and non-sodium glutamate or nucleotide systems while matching intensity, pH, and osmolality. Direct comparisons of MSG with potassium, calcium, or other glutamate salts should control intensity, ionic strength, concentration, pH, and food matrix; lower liking of a non-sodium salt would not support sodium priority unless these sensory and physicochemical differences were adequately controlled. Parallel studies of sodium containing and non-sodium inosinate/guanylate systems could test whether sodium contributes to liking, reinforcement, or intake independently of umami synergy. Athletes or other groups with controlled acute sodium loss may provide ethically feasible models. Human receptor and psychophysical studies should determine the relative contribution of amiloride-sensitive, amiloride-insensitive, and chloride-dependent pathways across food-relevant concentrations. TMC4 findings require direct evaluation in human tissue and perception rather than inference from mice. Neuroimaging and electrophysiology could test whether internal sodium state changes central responses to sodium containing versus non-sodium but perceptually matched stimuli.

Food studies should combine sensory, instrumental, and chemical measurements. Texture, sodium release, volatile generation and release, microbial stability, and temporal perception should be measured when NaCl is reduced or replaced. Longitudinal studies can determine whether gradual reduction changes preference for salt levels, whether those changes generalise across foods, and whether glutamate–sodium discrimination predicts future intake rather than merely correlating with current diet.

The most informative outcomes are choice, intake, reinforcement, and adaptation, not intensity ratings alone. Studies should preregister what results would support, refine, or reject the hypothesis. This approach would prevent the framework from becoming unfalsifiable and would distinguish a genuinely sodium-related behavioural effect from generic flavour enhancement.

## 16. Implications for Sodium Reduction, Dietetics, and Public Health

The hypothesis is clinically and publicly relevant because excessive sodium intake contributes to elevated blood pressure and cardiovascular risk, while global intake remains above recommended levels in many populations [6,7,8].

Sodium-reduction strategies should address multiple sensory and technological functions. Gradual reduction, spatial distribution of salt, aroma enhancement, herbs, spices, acids, texture design, potassium substitution, and appropriate umami-rich ingredients can help maintain acceptance [9,10,35]. However, replacing NaCl with MSG or sodium-containing flavour enhancers must be evaluated with regard to total sodium content rather than ingredients’ names. Potassium substitution can be valuable but requires management of bitterness, metallic notes, and clinical suitability.

Dietetic guidance should recognise adaptation and food context. Education is important, but consumers cannot compensate easily when much sodium is incorporated before purchase. Population reformulation and supportive food environments remain essential. The framework may help explain why some products are difficult to reformulate, but public health recommendations should continue to prioritise reduced exposure to excessive sodium.

## 17. Conclusions

Sodium is essential, continually lost, and homeostatically regulated. NaCl provides the dominant dietary route to saltiness, although human salt taste transduction remains mechanistically unresolved and may involve both sodium- and chloride-dependent pathways. Sodium salts also alter selected taste mixtures and food properties, but these effects are concentration-, stimulus-, and matrix-dependent.

The Sodium Priority Hypothesis proposes that physiological need and several sensory routes may converge to support the acceptance or acquisition of sodium-containing foods. It does not claim that sodium causes umami, that prehistoric populations universally added salt, or that sodium priority alone explains modern overconsumption. Sodium-linked umami is the most speculative component, and its evolutionary significance is uncertain because free glutamate is limited in many natural protein-rich foods.

The framework should therefore be regarded as a conceptual and falsifiable organising proposal, not a demonstrated biological model. Direct comparisons of sodium and non-sodium stimuli, controlled human sodium state studies, human receptor investigations, and food matrix experiments are required to validate, refine, or reject it. The hypothesis is explanatory rather than prescriptive and is consistent with the public-health need to reduce excessive sodium intake.

## Figures and Tables

**Table 2:** Evidence domains supporting, qualifying, or limiting the Sodium Priority Hypothesis.

Evidence Domain	Evidence Base/Population	Established Finding	Strength and Consistency	Relevance to Hypothesis	Main Limitation and References
Sodium physiology	Human physiology; animal mechanisms	Sodium is essential, extracellular, continually lost, and tightly regulated.	High.	Supports sodium as a nutritional target.	Essentiality alone does not prove sensory priority [2,3,4,5].
Sodium appetite	Rodent depletion; limited human intervention	Depletion robustly increases salt seeking in animals and can alter salt preference in humans.	High in animals; moderate/variable in humans.	Supports state-dependent motivation.	Most work uses NaCl; generalisation to complex foods is unknown [3,4,5,13,14,37,38].
Low-sodium cultures	Human observational / ethnographic physiology	Humans can maintain balance at very low habitual sodium intakes through conservation.	Direct but population-specific.	Shows that discretionary salt is not universally required.	Cannot reconstruct all prehistoric diets or selection pressures [6,36].
Salt-taste transduction	Mouse genetics; human tissue and psychophysics	Mouse ENaC pathway is established; human mechanisms are largely amiloride-insensitive and unresolved.	High for mouse ENaC; preliminary for human molecular pathways.	Defines the direct NaCl route and a major boundary.	TMC4 evidence is mainly murine; human sodium versus chloride contributions remain uncertain [11,12,15,16,17,18].
Potassium contrast	Human sensory and food-model studies	KCl is generally less salty and more bitter/metallic; sodium and potassium can differ in mixture effects.	Moderate.	Argues against simple liking of all essential cations.	Side tastes and concentration confound sodium-specific conclusions [9,20].
Counter-ion effects	Human psychophysics	Sodium salts vary in taste according to the anion, pH, and concentration.	Moderate to high.	Supports counter-ions as modifiers of sodium delivery.	Limited matched food-matrix studies [9,18,19].
Bitterness suppression	Human psychophysics; receptor assays	NaCl suppresses selected bitter stimuli and receptor–agonist responses.	Moderate; stimulus-specific.	Provides a plausible food-balancing route.	Not universal and may be central or peripheral [20,21,22,23,24].
Other taste mixtures	Human psychophysics	NaCl interacts with sweetness, sourness, and umami, often through mixture suppression.	Moderate; heterogeneous.	Broadens the framework beyond bitterness.	Not all interactions improve acceptance [19,25].
MSG and umami	Human sensory; receptor and animal neural evidence	Glutamate and nucleotides generate umami; MSG contains sodium.	High for umami; low for sodium-specific behaviour.	Motivates a future test of a sodium-linked savoury route.	Na^+^ is not required for umami synergy; matched non-sodium comparisons are scarce [26,27,28,29,30,31,32,33,34].
Food-matrix functions	Food science and sensory studies	Salt affects texture, volatile release, preservation, and sodium release.	High for technological effects.	Explains why sodium reduction can alter acceptance.	Changes may be unrelated to sodium-specific taste [10,35].
Modern overconsumption	Population evidence and food environment	Average intakes exceed recommendations in many populations.	High.	Creates translational relevance and potential mismatch.	Availability, learning, and food structure may explain excess independently [6,7,8,9,10].

**Table 3:** Comparison of existing explanatory frameworks with the Sodium Priority Hypothesis.

Framework	Primary Question	Established Contribution	What It Does Not Integrate	Contribution or Status of Sodium Priority Hypothesis
Homeostatic sodium appetite models	How does sodium deficit generate conservation and motivated intake?	Explains endocrine, neural and behavioural responses to deficiency.	Does not usually integrate multiple food-sensory routes or food reformulation.	Uses sodium appetite as physiological context; does not replace it.
Salt-taste transduction models	How are NaCl and other salts detected peripherally and centrally?	Identifies ENaC and other salt pathways, concentration effects and aversion.	Does not by itself explain complex-food acceptance or modern intake.	Treats salt taste as one route and explicitly acknowledges unresolved human mechanisms.
Umami/protein-related models	How do glutamate and nucleotides generate savoury taste and potentially signal foods?	Explains glutamate receptors and nucleotide synergy.	Does not test whether sodium bearing and non-sodium umami stimuli differ behaviourally.	Adds a testable, explicitly speculative sodium-linked behavioural comparison.
Taste-mixture and flavour-integration models	How do tastes, aromas, texture, and context combine in food perception?	Explains suppression, enhancement, learning and multisensory acceptance.	Usually not organised around a nutrient specific homeostatic target.	Asks whether selected mixture effects converge specifically on sodium containing foods.
Food-technology sodium-reduction models	How can sodium be reduced while maintaining safety, structure, and liking?	Explains gradual reduction, substitution, distribution and matrix design.	Does not necessarily connect reformulation difficulty to sodium appetite or evolutionary context.	Provides an explanatory bridge while recognising matrix effects as alternative mechanisms.
Sodium Priority Hypothesis	Do physiological need and several sensory routes converge to support sodium acquisition or acceptance?	No new mechanism is claimed as established.	Cannot be considered validated without direct comparative tests.	A conceptual, falsifiable synthesis that may be supported, narrowed, or rejected.

## Data Availability

No new data were created or analysed in this review. Data sharing is not applicable.
